# Epigenetic Dysregulations in Merkel Cell Polyomavirus-Driven Merkel Cell Carcinoma

**DOI:** 10.3390/ijms222111464

**Published:** 2021-10-24

**Authors:** John Charles Rotondo, Chiara Mazziotta, Carmen Lanzillotti, Mauro Tognon, Fernanda Martini

**Affiliations:** 1 Department of Medical Sciences, University of Ferrara, 44121 Ferrara, Italy; mzzchr@unife.it (C.M.); lnzcmn@unife.it (C.L.); mauro.tognon@unife.it (M.T.); 2 Center for Studies on Gender Medicine, Department of Medical Sciences, University of Ferrara, 64/b, Fossato di Mortara Street, Ferrara 44121, Italy; 3 Laboratory for Technologies of Advanced Therapies (LTTA), University of Ferrara, 44121 Ferrara, Italy

**Keywords:** Merkel cell polyomavirus (MCPyV), Merkel cell carcinoma (MCC), epigenetics, virus-driven tumors, histone posttranslational modifications, HPTMs, DNA methylation, microRNA, miRNA

## Abstract

Merkel cell polyomavirus (MCPyV) is a small DNA virus with oncogenic potential. MCPyV is the causative agent of Merkel Cell Carcinoma (MCC), a rare but aggressive tumor of the skin. The role of epigenetic mechanisms, such as histone posttranslational modifications (HPTMs), DNA methylation, and microRNA (miRNA) regulation on MCPyV-driven MCC has recently been highlighted. In this review, we aim to describe and discuss the latest insights into HPTMs, DNA methylation, and miRNA regulation, as well as their regulative factors in the context of MCPyV-driven MCC, to provide an overview of current findings on how MCPyV is involved in the dysregulation of these epigenetic processes. The current state of the art is also described as far as potentially using epigenetic dysregulations and related factors as diagnostic and prognostic tools is concerned, in addition to targets for MCPyV-driven MCC therapy. Growing evidence suggests that the dysregulation of HPTMs, DNA methylation, and miRNA pathways plays a role in MCPyV-driven MCC etiopathogenesis, which, therefore, may potentially be clinically significant for this deadly tumor. A deeper understanding of these mechanisms and related factors may improve diagnosis, prognosis, and therapy for MCPyV-driven MCC.

## 1. Introduction 

Merkel cell polyomavirus (MCPyV) is a DNA virus with oncogenic potential [1]. MCPyV is the causative agent of Merkel cell carcinoma (MCC) [2,3], a rare but aggressive neuroendocrine carcinoma of the skin [4,5]. About 80% of MCC cases are caused by MCPyV infection, while the rest do not harbor MCPyV DNA and/or proteins and are caused by UV-induced tumorigenic point mutations [6,7]. The expression of the two viral oncoproteins large T (LT) and small T (sT) antigens, alongside MCPyV DNA integration into the host genome, are responsible for driving tumorigenesis in MCPyV-induced MCC. Indeed, very few somatic mutations [8] and genomic rearrangements [7,9] have been found in MCPyV-driven MCCs compared with MCPyV-negative MCCs [2,6,10].

Epigenetics is a heritable, reversible condition with a role in controlling gene expression without altering DNA sequences. Epigenetic gene regulation occurs by histone posttranslational modifications (HPTMs), DNA methylation, and microRNA (miRNA) expression. These epigenetic mechanisms are connected, occurring in concert with other molecular processes/factors inducing chromatin architecture modifications, ultimately leading to gene regulation [11]. Alterations in HPTMs, DNA methylation, and miRNA expression, result in instable cell states; gene expression changes; and, eventually, carcinogenesis [12,13,14,15,16]. 

Epigenetic altering factors include oncogenic viruses, such as high-risk human papillomaviruses (HR-HPVs), Epstein–Barr virus, Hepatitis viruses, and Kaposi’s sarcoma-associated herpesvirus [17,18,19]. These viruses have been found to be associated with epigenetic dysregulations in different tumors [20,21,22], whereas their oncoproteins have been shown to induce dysfunctions in proteins/enzymes involved in controlling DNA methylation, chromatin modification, and miRNA expression [17,18,23]. Notably, epigenetic dysregulations have been shown to function as potential diagnostic and prognostic biomarkers as well as therapeutic targets in different virus-driven cancers [19,21]. 

Notwithstanding many investigations carried out in MCPyV-driven MCC, the role of epigenetic dysregulations in the onset of this tumor and the potential clinical applications remain largely unknown. A comprehensive review on these topics is so far nonexistent; thus, a literature search is undertaken herein for epigenetic alterations such as HPTMs, DNA methylation, and miRNA expression in MCPyV-driven MCC. Specifically, data were collected from the reported studies to summarize and compare the state of research in this emerging field and provide insight into the usage of potential diagnostic and prognostic biomarkers as well as targets for MCPyV-driven MCC therapy.

### 1.1. Merkel Cell Polyomavirus: Genomic Organization and Oncogenic Activity

MCPyV genome is a circular DNA of about 5,400 base-pairs (bp) [24]. The genome encompasses three regulatory regions, known as the noncoding control region (NCCR) and the early and late regions [25,26]. NCCR consists of the viral DNA replication origin, and early and late promoters/enhancers [25,27]. The early and late regions regulate early and late gene expression, respectively [28]. The early genes encode for transcripts generated by alternative splicing, such as LT and sT, 57 kT, and ALTO [29,30,31]. The late genes encode for the viral capsid proteins (VP1 and VP2) and miRNAs (see below) [27,32,33,34]. 

LT and sT are key players for MCPyV-driven MCC carcinogenesis [35,36]. LT binds to and inactivates the tumor suppressor protein retinoblastoma (pRB) [37], thereby leading to a proliferation maintenance in MCC cells [38,39], while sT bears transforming abilities [40]. In addition, a unique domain of sT, known as LT stabilization domain, confers the ability to bind to several tumor suppressor genes (TSGs), including FBW7, β-TrCP, and CDC20 to this oncoprotein, thereby leading to enhanced MCPyV replication and oncogene activation [41]. Moreover, as with other tumor viruses [42,43,44], MCPyV DNA integration into the host cell genome plays an important role in MCC carcinogenesis. While in non-tumor conditions, MCPyV infects Merkel cells and/or skin and blood cells by maintaining its genome in episomal form [45,46], viral DNA integration leads to mutational/deletion events in the viral genome, which prompt carcinogenesis [47]. Indeed, different truncated LT forms have been identified following viral DNA integration [27,48]. 

### 1.2. Epigenetic Machinery

**Figure 1:**
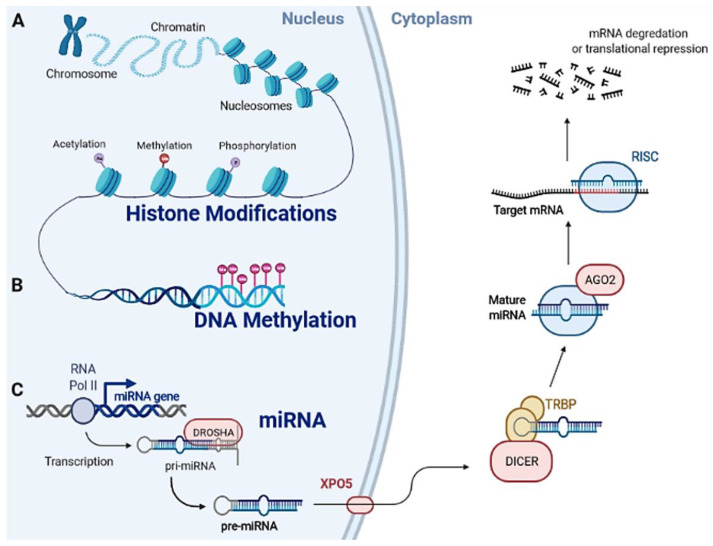
Epigenetic mechanisms. Epigenetic mechanisms comprise (**A**) histone posttranslational modifications (HPTMs), (**B**) DNA methylation, and (**C**) microRNAs (miRNAs) regulation. Gene expression can be regulated before transcription initiation by HPTMs and DNA methylation. Both mechanisms induce a remodeling of the chromatin structure, thereby making genes either less or more accessible for transcription factors, according to the different epigenetic modifications. Unlike DNA methylation and HPTMs, miRNAs regulate the expression of genes at the post-transcriptional level. miRNAs negatively regulate genes through complementing their mRNAs, which results in mRNA degradation or translational repression.

HPTMs, DNA methylation, and miRNAs are fundamental epigenetic mechanisms for controlling gene expression (Figure 1). 

HPTMs consist in covalent modifications of histones (Figure 1, panel A) [49,50]. The structure of histones H2A/H2B/H3/H4 is an octamer surrounded by 147 bp of DNA to form a nucleosome, which is the basic chromatin unit [51]. HPTMs predominantly take place on histone N-terminal regions (histone tails), occurring either alone or in combination, leading to gene expression regulation [52]. The combination of HPTMs with their regulatory role on gene expression is known as histone code, which may occur conjointly with DNA methylation [53]. Acetylation, methylation, and phosphorylation are the main HPTMs [54]. Histone acetylation provides an additional acetyl group to lysine, which, in turn, allows chromatin relaxation, making it accessible to transcription factors and RNA polymerase II [55]. This is understandable as acetyl groups on histone tails carry a negative charge, thus creating repulsive forces with the negatively charged DNA. Therefore, histone deacetylation induces chromatin condensation, which is linked to gene silencing [56]. The enzymes that add or remove the acetyl group are known as Histone Acetyltransferases (HATs) and Histone Deacetylases (HDACs), respectively [57]. Histone methylation occurs on lysine, which can be mono-/di-/tri-methylated, and on arginine, which can be mono-/di-methylated. Histone methylation is mediated by histone methyltransferases and, in general, correlates with transcriptional repression [58]. Histone demethylases catalyze demethylation reactions [59]. An important enzyme is Lysine-specific demethylase 1 (LSD1), which removes mono-/di-methylation marks on lysine 4 and/or 9 in histone H3 [60,61]. Methylated H3K9 is linked to epigenetic repression of heterochromatin [62], while methylation at H3K4 has been found in transcriptionally active euchromatic regions [63]. Histone phosphorylation provides additional phosphate groups to serine, threonine, or tyrosine [64,65,66]. Kinases and phosphatases are the two enzymes responsible for adding and removing phosphate groups from histone tails, respectively [67,68]. Histone phosphorylation appears to be positively related to gene expression as it leads to chromatin relaxation. Impairment of HPTMs/modifying enzymes is associated with cancer [69]. 

DNA methylation refers to the enzymatic transfer of a methyl group to the 5-carbon position on a nucleotide, usually a cytosine (Figure 1, panel B) [70]. This process predominantly occurs in CpG dinucleotides [71]. Regions rich in CpGs are typically proximal to regulatory regions, e.g., promoters [70,72,73,74], which regulate gene expression [75]. When methylated, these regions usually lead to gene silencing [70]. CpG methylation patterns are established by DNA methyltransferases (DNMTs) [76,77], including DNMT1/-3A/-3B/-3C [78,79]. DNMT1, also known as maintenance DNMT, copies pre-existing methylation marks onto new strands following DNA replication [79]. DNMT3A and DNMT3B are de novo DNMTs, as they are able to methylate previously unmethylated DNA sequences. DNMT3C selectively methylates the promoters of retrotransposons [77]. CpGs can be demethylated through processes that can be either (i) passive, occurring in replicating cells by preventing methyl groups from being added to the newly replicated DNA strands [80]; (ii) active, as mediated by ten-eleven translocation (TET) enzymes (TET1/-2/-3) [81,82]. DNA methylation is one of the most highly studied epigenetic modification in normal and cancer cells [72,83,84]. In physiological conditions [85], it assures proper gene expression, while its dysregulation can lead to diseases [86,87]. TSG silencing occurs as a consequence of improper hypermethylation of their promoters [88,89], thereby inducing the dysregulation of a variety of pathways, including cell growth, migration, and apoptosis, leading to tumorigenesis [90,91]. Genome-wide hypomethylation can induce genomic instability, promoting cell transformation as well [71,92].

miRNAs are small, single-stranded molecules (18–22 nucleotides) mediating the post-transcriptional regulation of gene expression (Figure 1, panel C) [93]. miRNA coding genes are mainly located within intergenic regions and introns of protein-coding genes [94]. miRNA biogenesis is mediated by RNA polymerase II, which transcribes an immature RNA containing one to six miRNA precursors (pri-miRNA) [95]. Soon after its synthesis, the pri-miRNA undergoes both capping and polyadenylation [96]. Then, pri-miRNAs are cleaved by the class 2 ribonuclease III Drosha in order to generate a pre-miRNA molecule, which is afterwards exported from the nucleus to the cytoplasm by exportin 5 (XPO5) and processed by Dicer, in complex with TAR RNA binding protein (TRBP), to generate a short RNA duplex (41–180 nucleotides) [97]. Only one strand of this duplex is incorporated into the RNA-induced silencing complex (RISC), whose key components are proteins of the Argonaute (AGO) family. Functionally active RISCs act as post-transcriptional gene regulator complexes, by binding mRNA targets to complemental incorporated miRNA, in order to carry out gene silencing [97]. miRNAs regulate crucial cellular processes, including metabolism, proliferation, differentiation, migration, apoptosis, and stress response [98]. Impairment of the miRNA regulative network can lead to disease development, including cancer [99,100]. miRNAs are commonly considered regulators of a variety of cancer-related pathways by targeting both oncogenes and TSGs [101]. 

## 2. Methods

We performed an investigation into the scientific literature by searching through the PubMed (Medline, https://pubmed.ncbi.nlm.nih.gov/, accessed date: 1 August 2021) database until August 2021. The studies describing epigenetic dysregulations in MCPyV-driven MCC from 2008 up to August 2021 were reviewed for specific topic areas and included, described, and discussed in this review. A total of 34 relevant articles were identified in literature using the keywords Merkel cell polyomavirus, MCPyV, Merkel cell carcinoma, MCC, large T antigen, LT, small T antigen, sT, epigenomics, epigenetics, (de)methylation, histone posttranslational modification, HPTM, (de)acetylation, phosphorylation, microRNA, and miRNA. Additional 54 articles were selected using the combinations of keywords such as Merkel cell polyomavirus and (1) epigenomics, (2) epigenetics, (3) (de)methylation, (4) histone posttranslational modification, (5) HPTM, (6) (de)acetylation, (7) phosphorylation, (8) microRNA, and (9) miRNA. In addition, figures included in this review were made by using the BioRender online tool (www.biorender.com, accession dates: 1 February 2021–1 August 2021).

## 3. Epigenetic Dysregulations in Merkel-Cell-Polyomavirus-Driven Merkel Cell Carcinoma

### 3.1. Aberrant Epigenetic Modifications in Merkel-Cell-Polyomavirus-Driven Merkel Cell Carcinoma

Histone posttranslational modifications (Table 1). The contribution of H3 lysine 27 trimethylation (H3K27me3) to MCPyV-driven MCC was investigated. H3K27me3 is a repressive mark established by the catalytic enzymatic subunit enhancer of zeste homolog 2 (EZH2) of the Polycomb Repressive Complex 2 (PRC2), while H3K27me3-induced TSGs silencing is a mechanism for tumor development [15,102]. The involvement of EZH2 in tumor development and progression has been investigated in different tumors, including MCPyV-driven MCC [103,104,105]. Specifically, the histone methyltransferase EZH2 has been found to be overexpressed in MCC tissues compared with normal skin specimens, with no differences between MCPyV-positive and -negative MCCs [105].

A proteome analysis of MCPyV-positive MCC cells identified 185 differentially expressed proteins, including proteins from all 5 histone families, with 15 different subfamily members such as H2A1-H [112]; this study highlighted a role for histone variants in MCC onset [112]. However, the potential HPTMs of these variants as well as their implication in MCPyV-driven MCC remain to be determined.

DNA methylation (Table 2). A study conducted on MCC biopsies reported *RASSF1A* hypermethylation in about half of cases [113]. Notably, although most samples were positive for MCPyV DNA, no correlation between *RASSF1A* hypermethylation and MCPyV was determined, nor has a statistical association been found between hypermethylation of the *cyclin-dependent kinase inhibitor 2A (CDKN2A)* gene promoter and MCPyV-positive MCCs [113]. As RB protein is the main target for MCPyV LT and plays a key role in MCPyV-driven MCC onset [37], genetic and epigenetic features of the *RB* gene have been studied in MCPyV-positive and MCPyV-negative MCC cases [114]. Promoter methylation has been found in all MCCs despite MCPyV DNA presence, and RB expression and survival [114], suggesting that *RB* methylation might occur independently of MCPyV-positivity in MCC.

A recent high-throughput study investigated the DNA methylation age in MCPyV-positive and -negative MCC tissues and in four MCPyV-positive MCC cell lines [120]. DNA methylation age resulted as lower compared with chronological age and independent of MCPyV in MCC tissues. Two MCC cell lines presented as epigenetically younger compared with their chronological age. Since low DNA methylation age might indicate stemness/pluripotency, cell pluripotency status was assessed, showing its absence in both MCC tissues and MCPyV-positive cell lines [120]. These data indicate that MCC seems to be characterized by both epigenetic youth and lack of pluripotency—independently, however, from MCPyV-positivity. 

Few additional studies have investigated promoter methylation in association with gene expression in MCC. A study conducted on primary and metastatic MCC tissues with unknown MCPyV-positivity and in six MCPyV-positive MCC cell lines investigated the mRNA/protein levels and methylation status of *O6-methylguanine-DNA methyltransferase (MGMT)* [117]. MGMT is an enzyme implicated in DNA repair and apoptosis. Analyses have revealed highly heterogeneous MGMT mRNA and protein expression levels in both MCC tissues and cell lines, whereas hypermethylation was found in two out of six MCPyV-positive cell lines [117]. Similar results have also been obtained for a gene involved in the *Hedgehog (Hh*) signaling pathway, known as *HH receptor Patched 1 (PTCH1*) [118]. The Hh pathway plays a role in embryogenesis and Merkel cell development/differentiation, while its reactivation in adulthood might contribute to cancer [118]. *PTCH1* has been studied in MCC biopsies and cells and basal cell carcinoma (BCC) tissues in relation to mRNA/protein expression, and DNA mutation and methylation. No differences in PTCH1 expression between MCCs and BCCs were determined, whilst both differed from healthy skin. Notably, *PTCH1* was found to be hypomethylated in MCC tissues and cell lines independently from its expression levels and MCPyV status [118]. 

microRNA. In recent years, several miRNAs have been described as dysregulated in MCPyV-driven MCCs (Table 3). Among these, *miR-375* has been studied the most [32,122,123,124,125,126,127,128]. The dual role of *miR-375* in cancer has been frequently remarked upon as it appears to act as either a tumor suppressor or oncomiR, depending on the tumor type [129]. Indeed, both tumor-promoting and tumor-suppressing properties have been attributed to this miRNA in different cancer types [129,130,131,132,133,134]. The role of *miR-375* in MCC is still unclear [32]. Its dysregulation has been determined for the first time in differentiated MCPyV-positive/-negative MCCs and BCCs, which possess overlapping histologic features but distinct cellular origins [126]. *miR-375* levels were found to be 60-fold higher in MCC than in BCC, and normal skin tissues (controls). Moreover, a significant overexpression of *miR-375,* together with *miR-30a*, *miR-30a-3p, miR-30a-5p, miR-34a, miR-769-5p, miR-142-3p*, and *miR-1539*, and *miR-203* downexpression, has been described in MCPyV-positive vs. MCPyV-negative MCCs [128,135]. On the contrary, *miR-375* levels resulted as similar in MCPyV-negative and MCPyV-positive MCC tissues/cells [126].

Finally, MCPyV expresses two miRNAs, known as *MCPyV-miR-M1-5p* and *-3p*, which are encoded by the late region and are able to negatively regulate LT expression [32,137,138]. *MCPyV-miR-M1-5p* is expressed at low levels in 50% of MCPyV-positive MCCs, while it has also been predicted to target genes playing a role in promoting immunity evasion and regulating viral DNA replication [139,140]. This miRNA is required for establishing a long-term, persistent viral infection [137].

### 3.2. Role of Merkel Cell Polyomavirus (MCPyV) Oncoproteins in the Epigenetic Dysregulation of MCPyV-Driven Merkel Cell Carcinoma

An important role for MCPyV-driven MCC onset through the induction of host cell epigenetic dysregulations can be attributed to the viral oncoproteins, which have been investigated functionally in several epigenetic processes, such as HPTMs and miRNA expression (Table 2 and Table 3). 

Attention has been given to histone methylation and LSD1 [108], which mediates methylation marks at H3K4/K9, with the aim of developing novel anti-MCC therapies [8,109,141]. During MCPyV-driven carcinogenesis, improper activation of LSD1 has been observed [109]. Indeed, sT is capable of recruiting the MYC homolog MYCL and its heterodimeric partner MAX to the EP400 subunit of the HAT complex [108], which, in turn, promotes the expression of downstream genes, including LSD1 [109]. By using an in vitro pharmacological screen to detect epigenetic regulators in MCC, a recent study has identified LSD1 as a pivotal enzyme in tumor grown both in vitro and in vivo and, therefore, a potential therapy target [8]. LSD1 targeting can drive MCPyV-positive MCC cells towards normal Merkel cell fate and induce cell cycle arrest and cell death in vitro, while it can counteract tumor growth in vivo. Hence, these findings describe LSD1 inhibition as a novel therapeutic strategy for MCPyV-driven MCC and have opened the way for new anticancer approaches [8,108,109,141,142].

The involvement of histone methylation and phosphorylation in MCPyV-driven MCC has been investigated in the context of MCPyV sT-related DNA damage response (DDR) activation [111]. Functionally, in vitro sT overexpression can prompt (i) phosphorylation of H2AX, a histone-associated DNA damage marker; (ii) dimethylation of H3 lysine 4 and H4 lysine 20 (H3K4me2/H4K20me2); (iii) phosphorylation/activation of DDR signaling/ATM downstream proteins. These data not only underline a connection between MCPyV sT and the DDR pathway, but also provide insight into how histone methylation/phosphorylation contributes to MCC carcinogenesis [111].

Functional experiments have been conducted for evaluating the implication of miRNA dysregulation in MCPyV-driven carcinogenesis. A previous study indicated that *miR-375* knockdown in MCPyV-positive MCC cells is unable to perturb cell viability, proliferation rate, or morphology [123]. One possible explanation is that these cells may depend on MCPyV LT/sT for sustained growth and survival, unlike MCPyV-negative cells, whose proliferation might be driven by *miR-375,* as suggested in an additional study [127]. Contrariwise, functional data also indicated that MCPyV LT induces *miR-375* expression upon *Antigen-Induced Atonal Homolog 1 (ATOH1)* expression [123], the chief regulator of Merkel cell development. Of note, *ATOH1* resulted similarly expressed in MCPyV-positive/-negative MCC cells, suggesting *miR-375* expression as a common event in the development of both MCPyV-positive and -negative MCC subtypes [7]. Other studies have suggested a dual role for *miR-375* in MCPyV-positive and MCPyV-negative MCC [143]. Indeed, functional experiments have indicated that *miR-375* expression inhibition and *miR-375* ectopic expression in MCPyV-positive and MCPyV-negative MCC cells, respectively, can decrease cell growth and migration, while prompting apoptosis and cell cycle arrest [143]. Additional functional experiments conducted in MCC cells indicated that (i) LT/sT expression upregulates *miR-375*; and (ii) *miR-375* targets two autophagy genes, including *Autophagy Related 7 (ATG7)* and *Sequestosome-1/ubiquitin-binding protein p62 (SQSTM1/p62)* [124]. Likely, despite conflicting results [144], *miR-375* might function as a oncomiR in MCPyV-driven MCC while acting as a tumor suppressor in MCPyV-independent MCC. However, further studies are needed to clarify the role of *miR-375* in MCPyV-driven MCC.

The dysregulation of *miR-200c-141* and *miR-183-96-182* has been reported recently in MCPyV-positive MCC cell lines at different degrees of neuroendocrine differentiation and epithelial–mesenchymal transition (EMT) [136]. Increased *miR-200c-141* and *miR-183-96-182* expression, as well as hypomethylation of their gene loci, have been found to reduce the expression of EMT-related genes, in MCPyV-positive MCC cells [136]. These results demonstrate a connection between neuroendocrine characteristics and a lack of EMT in MCC cells with MCPyV. In vitro data from MCC cells indicated that LT/sT expression can upregulate these miRNAs, while *Beclin-1 (BECN1),* which plays a critical role in autophagy/cell death [145], has been identified as their target [124]. 

### 3.3. Epigenetic Dysregulations as Diagnostic, Prognostic, and Therapy Target Tools in Merkel Cell Polyomavirus-Driven Merkel Cell Carcinoma

Few studies assessed the clinical utility of altered HPTMs in MCPyV-driven MCC (Table 1). A recent study evaluating H3K27me3 in MCC tissues, stratified according to MCPyV status/morphological type, reported lower H3K27me3 levels in MCPyV-negative MCCs than in MCPyV-positive tumors [69]. Furthermore, H3K27me3 resulted as significantly lower in MCPyV-negative MCCs combined with squamous cell carcinoma than in MCPyV-positive/-negative pure MCCs or pure histologic MCCs (regardless of MCPyV status). However, the prognostic value of H3K27me3 was subsequently excluded due to a lack of association between this epigenetic mark and MCC patient outcome [69]. An opposing result has been obtained in MCPyV-positive and -negative MCCs with pure histological features, including primary and metastatic lesions, as well as a small number of combined squamous and neuroendocrine carcinomas [110]. The study described a reduction in H3K27me3 expression in MCPyV-positive MCCs and in MCCs with pure histologic features [110]. 

The major histocompatibility complex (MHC) class-I receptors expressed on virus-infected/malignant cell surfaces prompt the identification and eradication by CD8+ T cells as an adaptive immune response mechanism for MHC class-I expressing cells. Negative regulation of MHC class-I represents one of the strategies adopted by MCC to escape host immune-surveillance [107,146,147]. Loss of HLA class-I expression, a complex of proteins encoded by MHC class-I locus, has been described in MCC tissues with unknown MCPyV-positivity and in MCPyV-positive MCC cells [107]. This loss has been linked to a decreased expression in several chief components of the antigen processing machinery, including the Transporter associated with Antigen Processing 1 (TAP1) and TAP2, as well as low-molecular-weight protein (LMP) 2 and LMP7. In vitro data and mouse models have demonstrated that impairment of these genes is attributable to improper H3K9 deacetylation proximal to their regulatory regions. Consistently, treatment with the HDAC inhibitor (iHDAC) Vorinostat on MCPyV-positive MCC cells can restore acetylation of histones in HLA class-I promoters leading to re-expression of antigen processing machine components [107]. Similar effects have also been reported for Domatinostat, an oral iHDAC, in MCPyV-positive/-negative MCC cells [148]. Indeed, treatment with Domatinostat can induce distinct gene expression signatures in antigen processing/presentation, cell-cycle arrest and apoptosis, which occur, however, independently of MCPyV-positivity. Domatinostat can also prompt HLA class-I re-expression, thereby restoring the susceptibility of tumor cells to immune system recognition/elimination [148].

MHC class I chain-related protein (MIC) A and B are expressed upon cell transformation and act as kill me signals for natural killer (NK) cells, which are activated against tumor cells during innate immune response [149]. MICA and MICB expression have been reported as being completely absent in MCC cells, while being expressed in a minority of MCC tissues [106]. Loss of MIC expression in MCPyV-positive MCC cells has been found to be induced by improper H3K9 deacetylation at the MIC promoter, whereas MIC expression could be restored in vitro/in vivo by pharmacological inhibition of HDACs with Vorinostat [106]. 

The clinical application of improper DNA methylation marks in MCPyV-driven MCC is still remarkably poor (Table 2). A study that evaluated *p14^ARF^* promoter methylation status in MCPyV-positive and -negative MCC cases and in relation to MCC-patient clinical data reported no statistical association [115]. A recent analysis reported hypermethylation of *RASSF2/-5C/-10* genes ranging from 7-23% in MCCs and absent in normal tissues, while no correlation between *RASSFs* methylation status and MCC characteristics (primary vs. metastatic), or MCPyV-positivity, was found [116]. The DNA methylation profile has been recently investigated in MCPyV-positive and -negative primary and metastatic MCC tumors and cell lines compared with paired normal tissues [121]. Specific DNA methylation patterns exhibiting potential clinical relevance for MCC management and correlating with MCC onset, MCPyV gene expression, neuroendocrine features, and H3K27me3 status have been described [121]. In particular, hypomethylation at *Lysine Demethylase 6B (KDM6B),* a gene involved in the negative regulation of H3K27me3 [150,151], has been hypothesized to prompt KDM6B overexpression in MCPyV-driven MCC, ultimately leading to a global reduction of H3K27me3 [121].

A recent multicentre study evaluated *Programmed Cell Death protein 1 (PD-1*) gene for DNA methylation in a set of MCPyV-positive/-negative MCC tissues and its methylation status in relation to several clinicopathological parameters in MCC patients [119]. High levels of *PD-1* methylation were linked to higher overall mortality, while low *PD-1* methylation was related to MCPyV. Low levels of *PD-1* methylation have also been found to be linked to clinicopathological features related to MCPyV-positive MCCs, such as age >75 yrs, absence of immune cells, no PD-L1 expression by immune cells, as well as better prognosis [119]. 

Regarding the clinical significance of miRNAs in MCPyV-driven MCC (Table 3), only *miR-375* has been investigated repeatedly in several observational studies and suggested as a diagnostic marker [128,135,136], though with discordant results [126,143]. Moreover, two different studies have reported *mir-30a* as dysregulated in relation to MCPyV-positivity [128,135], thereby underling its potential reliability as a diagnostic marker for MCPyV-driven MCC.

## 4. Discussion and Future Perspectives 

This review has collected and summarized the current findings on those epigenetic dysregulations, including alterations in HPTMs, DNA methylation, and miRNA expression, which may play a role in MCPyV-driven MCC. 

**Figure 2:**
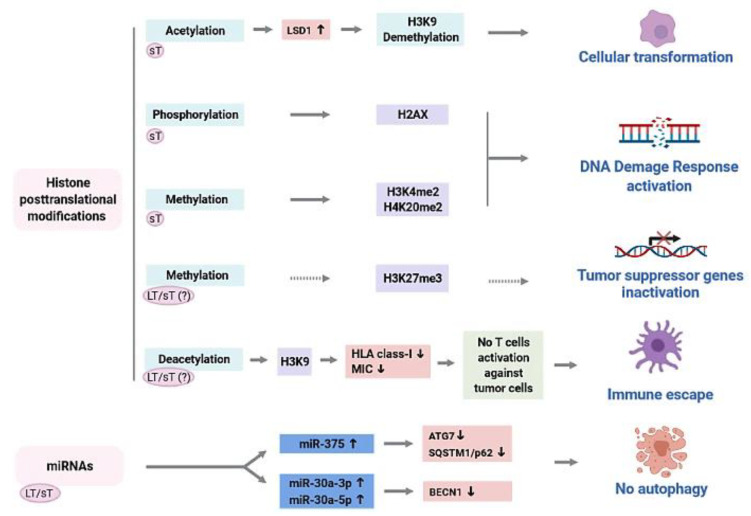
Impairment of epigenetic mechanisms in Merkel cell polyomavirus (MCPyV)-driven Merkel cell carcinoma. MCPyV sT promotes LSD1 expression through histone acetylation activation to induce cellular transformation. MCPyV sT can induce H2AX phosphorylation and H3K4 and H4K20 dimethylation (H3K4me2 and H4K20me2), thereby leading to the DNA damage response pathway activation. Tumor suppressor genes silencing via H3K27me3 mark might be a mechanism in MCPyV-driven MCC tumorigenesis. Loss of HLA class-I and MICA/B expression via H3K9 deacetylation might represent a strategy to evade the antiviral/-tumoral immune response. MCPyV LT/sT expression can upregulate miRNAs targeting genes involved in autophagy/cell death, such as *ATG7*, *SQSTM1/p62,* and *BECN1*. Continuous arrows—epigenetic dysregulation determined functionally in vitro. Dashed arrows—hypothesized epigenetic mechanisms. LT/sT (?)—the role of MCPyV LT/sT proteins has not been demonstrated.

Overall, the molecular mechanisms at the basis of epigenetic dysregulations in MCC onset, particularly in the context of MCPyV infection, are limited yet determined (Figure 2). 

It appears that different epigenetic marks are linked to MCC to some extent, while the direct involvement of MCPyV oncoproteins in these processes is de facto still unclear (Figure 2). Epigenetic dysregulations of the HMC locus through improper deacetylation of histones appear to be an antiviral/-tumoral immune response evasion strategy (Figure 2) [106,107]. Furthermore, MCPyV oncoproteins seem to be capable of epigenetically suppressing autophagy to protect cancer cells from cell death by inducing increase expression of *miR-375*, *miR-30a-3p*, and *miR-30a-5p* (Figure 2) [124]. In summary, it is becoming increasingly evident that in MCPyV-driven MCC, viral oncoproteins interact with a variety of cellular factors, including those involved in epigenetic pathways [124]. It is plausible that these mechanisms provide intricate interactions between viral and genetic/epigenetic players/pathways, which have only been partially identified to date [152]. Thus, further functional studies are required to understand how MCPyV is capable of dysregulating these epigenetic mechanisms, ultimately leading to MCC onset.

Despite a growing number of studies aimed at assessing whether epigenetic dysregulations might potentially improve MCPyV-driven MCC diagnosis, prognosis, and therapy, only a few have provided robust conclusions. 

Regarding the clinical application of dysregulated HPTMs, the most promising results have been obtained with two iHDACs—i.e., Vorinostat and Domatinostat, which restore the susceptibility of tumor cells to immune system recognition/elimination [107,148]. Moreover, identifying LSD1 as an important enzyme for MCPyV-driven MCC has opened the way for developing novel antitumor therapeutic strategies, as it can also be exploited as a potential therapy target [8]. Notably, the clinical application of these data has been shown as effective since these studies have been conducted not only in vitro with MCC cells, but also in vivo using animal models [8]. In addition, the aforementioned therapeutic approaches have also been described in treating a variety of solid tumors with significant results [153,154]. Inversely, slight and discordant information has been given regarding the diagnostic and prognostic application of HPTMs, such as acetylation/deacetylation, methylation/demethylation, and phosphorylation, in MCPyV-driven MCC. Further studies are therefore needed to identify HPTMs to be employed as helpful tools in MCPyV-driven MCC diagnosis and prognosis. 

The use of improper DNA methylation marks in MCPyV-driven MCC for clinical purposes is still poor. One study found an association between hypomethylation at *PD-1* gene in relation to patient outcome, when used as prognostic marker [119], while a lack of diagnostic significance has been reported for several candidate genes, such as *p14^ARF^/CDKN2A*, *MGMT*, *PTCH1,* and members of the *RASSF* family [113,115,116,117,118]. The potential diagnostic and prognostic utility of *KDM6B* gene hypomethylation in MCPyV-driven MCC management has also been pointed out [121]. No data have been reported on DNA methylation as a therapy target for MCPyV-driven MCC. In summary, current data suggest that applying defective DNA methylation as a diagnostic and prognostic tool for MCPyV-driven MCC is as yet unlikely, while the therapeutic application of DNA methylation remains to be determined.

The clinical significance of miRNA dysregulation in MCPyV-driven MCC also remains to be defined. Although conflicting results have been reported [140,151], only a few studies hypothesized a clinical application for *miR-375* as a diagnostic marker [128,135,136]. In addition, the current data available do not provide enough information on a possible clinical application for additional miRNAs being investigated in MCPyV-driven MCC [136]. Further studies in this field should therefore be performed. Evaluating the relationship between miRNAs and MCPyV might represent a promising future area of study aimed at identifying novel clinical options for diagnosis, prognosis, and treatment of this tumor [155]. 

## 5. Concluding Remarks 

Determining a link between MCPyV and epigenetic dysregulations upon MCPyV-driven MCC onset/development is of paramount significance to improve diagnostic, prognostic, and therapeutic options, as well as for a better understanding of the molecular mechanisms at the basis of this tumor. A clear grasp of how MCPyV oncoproteins might drive MCC onset/development, possibly involving epigenetic dysregulations, would help in identifying novel diagnostic and prognostic markers and in developing novel antitumor therapies [156].

It should be underlined that, when studying HPTMs, DNA methylation, and miRNA on MCC tissues from an observational point of view, the results might be susceptible to potential bias. For instance, a lack of statistical significance might be accounted for a reduced sample size [69,115]. Collecting large sets of tissues in order to reach statistical significance when comparing MCPyV-positive vs. -negative MCC tissues could be rather difficult, as MCC is a rare tumor [157]. To this end, multicenter studies based on large sample sizes should always be considered when conducting such epigenetic analyses on MCC tissues [119]. Extending these observational analyses to MCC cell lines is an additional option that should be taken into account, despite being only performed sparsely [117,126,128,136]. Regarding the study of DNA methylation and miRNA expression on MCC tissues, an additional point to be considered is the potential contamination by nontumor cells, such as blood, white, and endothelial cells [158]. Just a few contaminating nontumor cells could potentially modify DNA methylation/miRNA signatures of tumor cells, thereby hampering data. Establishing primary cultures derived from fresh MCC tissues might circumvent potential contamination of nontumor cells in order to provide more reliable information, as proposed for other virus-driven tumors [159].

In conclusion, understanding epigenetic mechanisms, players, and connections in order to improve diagnosis, prognosis, and therapy in MCPyV-driven MCC is promising yet challenging. Identifying novel robust epigenetic markers such as HPTMs, methylated DNA sequences/genes, and differentially expressed miRNAs may improve early diagnosis, patient monitoring, and therapy of virus-related diseases [160,161,162], including MCPyV-driven MCC. Investigations into the epigenetic mechanisms behind MCPyV-driven MCC represent a relevant future area of study. 

## Figures and Tables

**Table 1:** Histone posttranslational modifications (HPTMs) and/or HPTMs modifying enzymes in Merkel cell polyomavirus (MCPyV)-positive Merkel cell carcinoma (MCC) tissues and/or MCPyV-positive MCC-derived cell lines.

Modification	Histone	Site	Experimental Model	Enzyme	Reference
Deacetylation	H3	K9	MCPyV-positive MCC cell lines	-	[106]
Deacetylation	H3	K9	MCPyV-positive MCC cell lines and mouse models	-	[107]
Acetylation	-	-	MCPyV-positive/-negative MCC cell lines	MYCL and EP400 complex	[108]
Demethylation	-	-	MCPyV-positive MCC cell lines	LSD1	[109]
Demethylation	-	-	MCPyV-positive MCC cell lines	LSD1	[8]
Methylation (me3)	H3	K27	MCPyV-positive/-negative MCC tissues	-	[69]
Methylation (me1-2-3)	-	-	MCPyV-positive/-negative MCC tissues	EZH2	[105]
Low methylation (me3)	H3	K27	MCPyV-positive/-negative MCC tissues	-	[110]
Methylation (me2)	H3	K4	No-MCC cell lines expressing MCPyV sT antigen	-	[111]
Methylation (me2)	H4	K20	No-MCC cell lines expressing MCPyV sT antigen	-	
Phosphorylation	H2AX	S139	No-MCC cell lines expressing MCPyV sT antigen	-	

**Table 2:** Differentially methylated genes in Merkel cell polyomavirus (MCPyV)-positive Merkel cell carcinoma (MCC) tissues and/or MCPyV-positive MCC-derived cell lines.

Gene	Function	Promoter Methylation	Experimental Model	Reference
*P14^ARF^*	Tumor suppressor protein	Hypermethylated	MCPyV-positive/-negative MCC tissues	[115]
*CDKN2A*	Tumor suppressor protein	Hypermethylated	MCPyV-positive/-negative MCC tissues	[113]
*RASSF1A*	Tumor suppressor protein	Hypermethylated		
*RASSF2*	Tumor suppressor protein	Hypermethylated	MCPyV-positive/-negative MCC tissues	[116]
*RASSF5C*	Tumor suppressor protein	Hypermethylated		
*RASSF10*	Embryonic neurogenesis	Hypermethylated		
*RB1*	Tumor suppressor protein	Hypermethylated	MCPyV-positive/-negative MCC tissues	[114]
*MGMT*	DNA repair and apoptosis	Hyper-/Hypomethylated	MCPyV-positive MCC cell lines	[117]
		Hypomethylated	MCC tissues *	
*PTCH1*	HH receptor	Hypomethylated	MCPyV-positive/-negative MCC tissues	[118]
*PD-1*	Immune-inhibitory receptor	Hypomethylated	MCPyV-positive/-negative MCC tissues	[119]
Multiple genes		Hyper-/Hypomethylated	MCPyV-positive/-negative MCC tissues/cell lines	[120]
Multiple genes		Hyper-/Hypomethylated	MCPyV-positive/-negative MCC tissues/cell lines	[121]
*KDM6B*	H3K27 demethylation	Hypomethylated	MCPyV-positive tissues	

* Unknown MCPyV positivity.

**Table 3:** Differentially expressed miRNAs in Merkel cell polyomavirus (MCPyV)-positive Merkel cell carcinoma (MCC) tissues and/or MCPyV-positive MCC-derived cell lines.

miRNA↑	miRNA↓	Experimental Model	Reference
miR-375	→	MCPyV-positive/-negative MCC vs. non-MCC tissues and cells lines *	[126]
miR-375		MCPyV-positive vs. MCPyV-negative MCC cell lines	[127]
miR-200c-141 miR-183-96-182		MCPyV-positive vs. MCPyV-negative MCC cell lines	[136]
miR-30a-3p miR-30a-5p miR-375 miR-34a miR-769-5p	miR-203	MCPyV-positive vs. MCPyV-negative MCC tissues and cell lines	[128]
miR-30a miR-34a miR-142-3p miR-1539		MCPyV-positive vs. MCPyV-negative MCC tissues	[135]

* Lack of relationship between miRNAs dysregulation and MCPyV presence.

## Data Availability

Not applicable.
